# Bacsnp: Using Single Nucleotide Polymorphism (SNP) Specificities and Frequencies to Identify Genotype Composition in Baculoviruses

**DOI:** 10.3390/v12060625

**Published:** 2020-06-09

**Authors:** Jörg T. Wennmann, Jiangbin Fan, Johannes A. Jehle

**Affiliations:** Institute for Biological Control, Julius Kühn-Institut, Heinrichstraße 243, 64287 Darmstadt, Germany; fan.jiangbin@julius-kuehn.de (J.F.); johannes.jehle@julius-kuehn.de (J.A.J.)

**Keywords:** genome sequencing, genetic variability, sequence heterogeneity, dsDNA viruses, Baculoviridae, *Cydia pomonella* granulovirus

## Abstract

Natural isolates of baculoviruses (as well as other dsDNA viruses) generally consist of homogenous or heterogenous populations of genotypes. The number and positions of single nucleotide polymorphisms (SNPs) from sequencing data are often used as suitable markers to study their genotypic composition. Identifying and assigning the specificities and frequencies of SNPs from high-throughput genome sequencing data can be very challenging, especially when comparing between several sequenced isolates or samples. In this study, the new tool “bacsnp”, written in R programming langue, was developed as a downstream process, enabling the detection of SNP specificities across several virus isolates. The basis of this analysis is the use of a common, closely related reference to which the sequencing reads of an isolate are mapped. Thereby, the specificities of SNPs are linked and their frequencies can be used to analyze the genetic composition across the sequenced isolate. Here, the downstream process and analysis of detected SNP positions is demonstrated on the example of three baculovirus isolates showing the fast and reliable detection of a mixed sequenced sample.

## 1. Introduction

In recent years, sequencing of genomes of large dsDNA viruses has been facilitated by the availability of whole genome sequencing techniques that allow deciphering of genomes in relatively short time with up to thousands-fold read depth [1]. One of the most frequently applied methods is massive parallel sequencing techniques that are based on the fragmentation of genomic DNA, size selection of fragments and their amplification by PCR followed by massive parallel sequencing, resulting in millions of short sequenced fragments (reads) of a previously defined length. Nowadays, most dsDNA virus genome sequences published on GenBank have been determined by Illumina devices, in which read lengths can be set between 75 and 300 nt, and from which a consensus sequence can be assembled by bioinformatic workflows. One of the largest groups of dsDNA viruses are baculoviruses (family *Baculoviridae*, [2]), which are specific for insects from the orders Lepidoptera, Hymenoptera and Diptera. Their genomes are 80 to 180 kbp long and encode 100 to 200 open reading frames long [2]. More than 200 baculovirus genomes have been sequenced [3]. Since numerous baculoviruses are used as biological pest control agents, there is a considerable scientific and economic interest in the identification of isolates and the elucidation of genetic variability.

One applied technique to access genetic variation within sequenced baculovirus isolates or specimens is the detection of insertion and deletions (indels), as well as single nucleotide polymorphisms (SNP) [1,4,5,6]. In most cases, SNP positions are determined by using alignment of consensus sequences, previously generated from the same data [5,7], or the re-mapping of short-read sequencing data against their consensus sequence in order to evaluate intraspecific genetic variation within the sequenced baculovirus population [8,9].

More recent approaches used a combination of both methods, a determination of SNP specificities by consensus sequence alignments, and the use of those specific positions to quantify the presence of a certain isolate [6] or even use a single consensus sequence to compare occurring SNP patterns to draw conclusions about the relatedness and similarity of sequenced isolates [10]. However, very often the determination of SNP specificities by previously called consensus sequences can be considered as a bottleneck, since the establishment of a consensus sequence entails the risk of diminishing intraspecific variation at the first step. Although a consensus can indicate the presence of intraspecific variation by ambiguous nucleotides, the ratio of the present nucleotides in these positions remains hidden. The need to examine sequenced baculovirus samples for the presence of several genotypes was evident with the finding of clearly distinct but mixed genotypes. As an example, the Cydia pomonella granulovirus (CpGV) isolates 0006 (CpGV-0006) and R5 (CpGV-R5) can be named, which were found to be clearly distinguishable mixtures of two known and previously described isolates of CpGV with different biological characteristics [6], a conclusion that cannot be drawn from a consensus sequence.

For that reason, this study focused on establishing an analysis method that (i) avoids creating a consensus sequence from the available data and yet (ii) allows the detection of previously known as well as unknown SNP positions. The only requirement is the choice of a reference sequence that should be closely related to the analyzed samples, e.g., from the same species. On the example of three isolates of the *Cydia pomonella granulovirus* species (CpGV) (genus *Betabaculovirus*) [2], we describe how a consensus sequence free method can be used to detect isolate or specimen-specific SNPs positions and how those can be used to decipher the genotypic composition of isolates. The method follows a highly standardized workflow including a newly developed tool, termed bacsnp, written in R programming language, allowing the determination of SNP specificities across several sequenced isolates. For validation, the analysis was conducted independently with two CpGV reference sequences, one being closely related and one being more distantly related to the three analyzed samples in order to investigate the influence of the chosen reference sequence. In both cases, a mixed isolate composed of mainly two dominant genotypes was deciphered correctly. As demonstrated, the new tool bacsnp allows determining SNP specificities across several sequenced isolates, enabling insight into genetic variation at an intraspecific level.

## 2. Materials and Methods

### 2.1. CpGV Isolates

Three isolates of Cydia pomonella granulovirus (CpGV) were used: CpGV isolates CpGV-M (genome group A) and CpGV-S (genome group E) [11] were obtained from the virus collection at the Julius Kühn-Institut (JKI), Institute for Biological Control, in Darmstadt. These virus stocks were used for previous bioassay studies [12,13,14] and sequencing analysis [5,11]. The isolate CpGV-0006 derived from the commercial product MadexMAX (Andermatt Biocontrol, Stahlermatten, Switzerland) [6,15].

### 2.2. Genome Sequencing

The DNA of CpGV-M and CpGV-S was isolated according to laboratory standard protocol [6] and 100 ng high quality genomic DNA of each isolate was sent for sequencing performed by using NexteraXT library (Illumina Inc., San Diego, CA, USA) preparation and an Illumina NextSeq500 (Illumina Inc., San Diego, CA, USA) sequencing system (StarSEQ Ltd., Mainz, Germany). About 3.5 million paired-end reads of 151 nt in length were obtained for each isolate. The raw sequencing data of isolate CpGV-0006, which was sequenced previously accordingly with the same parameters [6], was included and re-analyzed in this study.

### 2.3. Raw Data Processing

**Figure 1:**
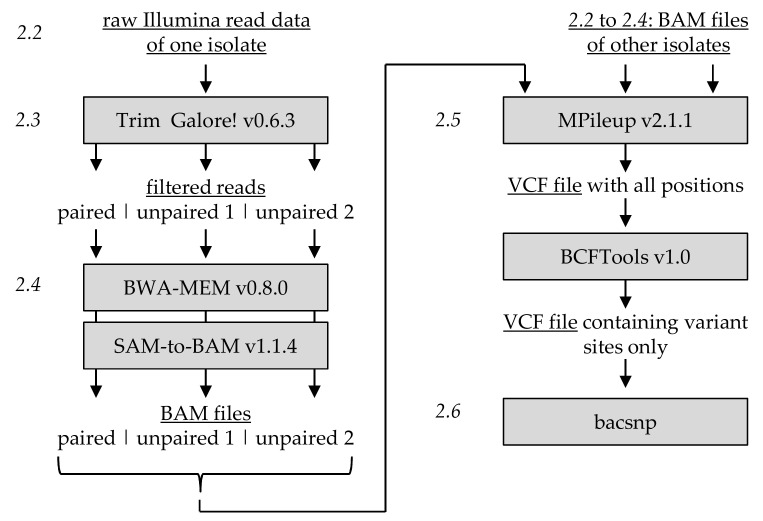
Workflow of processing Illumina sequencing data for the detection of variable single nucleotide polymorphisms (SNP) positions. Steps 2.2 to 2.6 refer to the corresponding paragraphs in the text. Steps 2.2 to 2.4 were applied separately for each sequenced isolate. All binary alignment mapping (BAM) files of all isolates were processed commonly using MPileup (step 2.5) to detect variant sites and to analyze their specificities and frequencies (step 2.6).

The raw Illumina read data of CpGV-M, CpGV-S and CpGV-0006 were uploaded on a JKI Galaxy server and were processed in exactly the same manner by using the Galaxy server‘s workflow tool and assigning unique group identifier for each isolate (Figure 1). Raw reads were adapter trimmed and quality filtered using Trim Galore! v0.6.3 [16] with a minimal Phred >30 and a minimal paired and unpaired read length of 50 and 51 nt, respectively, resulting in three groups of reads for each isolate: (i) unpaired forwards and (ii) unpaired reverse reads whose corresponding partner did not fulfill the quality and length criteria and (iii) quality filtered paired reads.

### 2.4. Mapping Against Common Reference Sequence

For each isolate, the unpaired and paired reads were mapped in three independent steps against the reference sequence CpGV-M (GenBank accession no. KM217575) using BWA-MEM v0.8.0 [17] (Figure 1) with default parameters resulting in three sequence alignment mapping (SAM) files. SAM files were transformed to binary alignment mapping (BAM files) using SAM-to-BAM v1.1.4 [18] (Figure 1). To investigate the influence of the reference sequence used for the analysis, step 2.4 was conducted independently using the English isolate CpGV-E2 (KM217577) (genome group B) as the reference sequence. CpGV-E2 was known not to be part of CpGV-M, CpGV-S and CpGV-0006, but also belongs to the *Cydia pomonella granulovirus* species [3].

### 2.5. Detection of Single Nucleotide Polymorphisms

For the detection of SNPs, the nine BAM files of CpGV-M, CpGV-S and CpGV-0006 were processed commonly using MPileup v2.1.1 [18] to call variant positions in correlation to the common reference genome CpGV-M (Figure 1). The output parameters were set to contain the number of high-quality bases (DP) and number of high-quality bases for each observed allele (DRP). Calling of insertions and deletions (indels) was not performed. The uncompressed variant file format (VCF) output was filtered using BCFTools v1.0 [18] with the following options: do not skip SNP positions, keep all alternative alleles and variant sites only. The final VCF file contained all detected SNP positions in relation to the reference CpGV-M (KM217575) for the sequenced and analyzed isolates CpGV-M, CpGV-S and CpGV-0006. For each SNP position, the total read depth as well as the count of reference and the three possible alternative nucleotides were saved in the output. Step 2.5 was repeated independently with the exact same parameters for the nine BAM files that resulted from step 2.4 with CpGV-E2 as reference.

### 2.6. SNP Specificity and Quantitative Analysis

To define the specificity of each SNP position, a de novo SNP calling approach was used that is provided by bacsnp package v0.1.0 developed in R programming language (v3.6.1) using RStudio (v1.2.5001) (Figure 1). The bacsnp package is available at the corresponding GitHub repository [19] and provides functions of all procedures described in the following protocol. Prior to assigning specificities of SNP positions to certain isolates, the absolute counts of reference and alternative nucleotides saved in the VCF data were transformed to relative frequencies. The SNP table containing positions in relation to CpGV-M, absolute counts and relative frequencies of reference and alternative nucleotides was filtered. To reduce background noise by sequencing errors and minor SNPs caused by assembly errors occurring in repetitive genome regions, e.g., repeat regions, the SNP data was filtered under the following criteria: only SNP positions at locations with an absolute total read depth >100 were considered, alternative read counts should be higher than 10, and the relative frequency (*ƒ*) should exceed *ƒ* > 0.05. The filtered SNP table was then conducted for specificity determination of SNP positions based on the alternative nucleotide. The assumption behind the de novo SNP specificity assignment is that a variable SNP position can possess three different frequencies (*ƒ*) for each isolate:

(i) *ƒ* = 0, identical to the reference sequence (but detected variable in another isolate and therefore detected in the first place),
(ii) *ƒ* = 1, entirely different to the reference sequence or
(iii) 0 < *ƒ* < 1, both nucleotides, identical and different to the reference, were found.

Based on these assumptions, SNP positions were assigned isolate or group (for more than one isolate) specific in R using the workflow provided by the bacsnp package under the following criteria:

(i) A SNP position is assigned as being solely specific for one isolate, if all other isolates do not show alternative nucleotides in the same position, or
(ii) is assigned as group specific if more than one isolate shows alternative nucleotides in this position.

Since CpGV-0006 was known to be a mixture of CpGV-M and CpGV-S [6], SNP specificities were only determined for CpGV-M and CpGV-S and then transferred to CpGV-0006 as provided by the bacsnp package. The ability to transfer SNP specificities of CpGV-M and CpGV-S to CpGV-0006 reflected the strength of the de novo approach due to the association of SNP positions linked to a common reference genome.

## 3. Results

For the detection of variable positions in sequenced isolates CpGV-M, CpGV-S and CpGV-0006, their quality filtered reads were mapped in two separate analyses against either CpGV-M or CpGV-E2 as a common reference. After filtering, most of the reads were still paired, while the unpaired reads were only between 3.8% and 4.3% (Table 1). Based on the paired reads alone, an entire coverage of the reference genome was given with read depths up to 3000 for CpGV-M and -S, which was ensured by the large proportion of reads that mapped to CpGV-M and CpGV-E2, respectively (Table 1).

By a commonly used reference, SNP positions were linked between isolates and an overall number of 284 and 322 SNP positions were found in the unfiltered SNP dataset when using CpGV-M and CpGV-E2 as reference, respectively. For each SNP position, four frequencies were obtained: the reference and the three possible alternative nucleotides. To determine how frequent more than one alternative nucleotide occurred within detected SNP positions, the mean frequencies of the reference, *ƒ*_ref_, and the three alternatives, *ƒ*_alt1_, *ƒ*_alt2_ and *ƒ*_alt3_, were calculated on the example of the 284 SNP positions that were found when CpGV-M was used as reference (Table 2).

The mean frequency (*ƒ*_ref_) of the reference nucleotide varied between 0.087 ± 0.252 and 0.97 ± 0.092 for CpGV-S and CpGV-M, respectively, whereas it was 0.394 ± 0.199 for CpGV-0006 (Table 2). For the first alternative nucleotide, the average frequencies (*ƒ*_alt1_) were approximating 1 − *ƒ*_ref_ (Table 2) and, therefore, both the frequencies of the reference and the first alternative (*ƒ*_ref_ + *ƒ*_alt1_) explained about 99.7% (CpGV-S) to 99.8% (CpGV-M and CpGV-0006) of the SNPs found (Table 2). A second or third alternative nucleotide was found for all three sequenced isolates only at average frequencies (*ƒ*_alt2/alt3_) between *ƒ*_alt2/alt3_ = 0.002 and 0.003 (Table 2). Since the occurrence and frequency of the second and third alternative appeared to contribute little to the entire data set, the assignment of SNP specificities was performed on the frequencies of the first alternative nucleotide only. When the above described average frequency analysis was performed for the unfiltered dataset using CpGV-E2 as reference, the sum of the average frequencies of the reference and the first alternative (*ƒ*_ref_ + *ƒ*_alt1_) explained 99.7% (CpGV-M) and 99.8% (CpGV-S and CpGV-0006), confirming to proceed with the SNP specificity determination with the first alternative.

**Figure 2:**
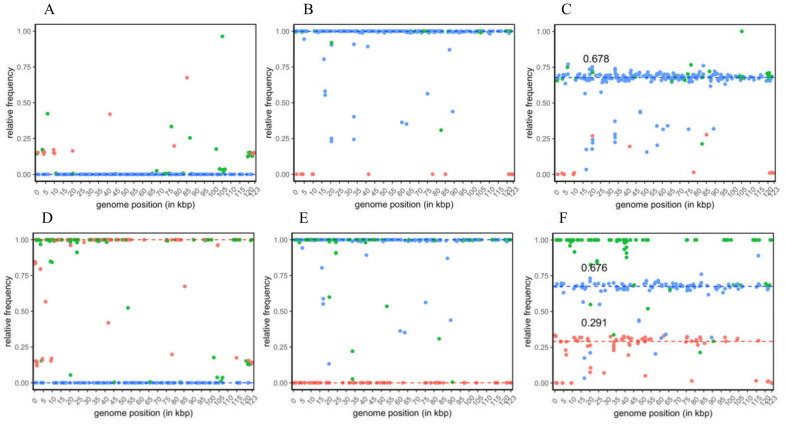
Single nucleotide frequency (SNP) plots of sequenced isolates CpGV-M (**A** and **D**), CpGV-S (**B** and **E**) and CpGV-0006 (**C** and **F**) mapped against reference sequences CpGV-M (KM217575) (**A**, **B** and **C**) and CpGV-E2 (KM217577) (**D**, **E** and **F**). For the CpGV-M and CpGV-E2 reference sequence-based analyses, 277 and 300 variable SNP positions were found, respectively, and the frequency of the alternative nucleotide was plotted (dots). The specificities of SNP positions were marked red for CpGV-M (*n* = 15 and *n* = 82 for CpGV-M and CpGV-E2 reference based analysis, respectively), blue for CpGV-S (*n* = 223 and *n* = 113 for CpGV-M and CpGV-E2 reference based analysis, respectively) and green for both isolates (*n* = 39 and *n* = 105 for CpGV-M and CpGV-E2 reference based analysis, respectively). Median frequencies for CpGV-M and CpGV-S are indicated by red and blue dashed lines, respectively; numbers indicate the median frequency of CpGV-M and CpGV-S-specific SNPs in CpGV-0006.

The raw SNP data sets were filtered to increase the stringency for further SNP specificity assignment and quantitative analysis. For the analysis based on CpGV-M and CpGV-E2 as references, the total number of SNP positions decreased to 277 and 300, respectively, of which most filtered SNP positions were only located in repeat regions of their reference. From the 277 SNP positions of the CpGV-M reference-based analysis, 223 were specific solely to CpGV-S, 15 were specific to CpGV-M, and 39 were group specific for both isolates (Figure 2A–C). For the majority of CpGV-S-specific SNP positions, their relative frequencies (*ƒ*_S_) grouped around three medians in the three analyzed isolates: (i) *ƒ*_S_ = 0 in CpGV-M (Figure 2A), (ii) *ƒ*_S_ = 1 in CpGV-S (Figure 2B) and (iii) *ƒ*_S_ = 0.678 in CpGV-0006 (Figure 2C). In the sequenced isolates CpGV-S and CpGV-0006, the frequencies of a minority of CpGV-S specific SNPs scattered below the calculated median (Figure 2B,C).

Interpretation of CpGV-M-specific SNPs in the three samples appears less obvious. Since the data of the re-sequenced CpGV-M sample was mapped against its own CpGV-M reference sequence (a previous consensus sequence) available at GenBank, only SNPs were expected that reflect either intraspecific variation within this isolate or sequencing errors in the reference sequence itself. From this self-mapping of CpGV-M reads, the relative frequencies of CpGV-M-specific SNP positions (*ƒ*_M_) ranged between *ƒ*_M_ = 0.136 and *ƒ*_M_ = 0.675 (Figure 2A), indicating an absence of large genotypic variation within the CpGV-M sample. This finding was supported by the relative frequencies of the group-specific SNP positions (*ƒ*_M+S_) that varied between *ƒ* = 0.004 and *ƒ* = 0.962 (Figure 2A). Based on the observations made by the self-mapping of CpGV-M against its own reference, its high homogeneity was assumed based on the following three assumptions: The (i) median frequency of the CpGV-S-specific SNP positions were *ƒ*_S_ = 0 in CpGV-M, (ii) the CpGV-M-specific SNP positions could only reflect its own intraspecific variation, and (iii) any large genetic variation was not detected (only 15 CpGV-M-specific SNP positions and relative frequencies *ƒ*_M_ < 0.675) (Figure 2A).

For CpGV-S, a different situation arose since the reference sequence CpGV-M was more distantly related to the sequenced isolate. This led to the detection of the 223 CpGV-S-specific SNP positions, whose median frequency was *ƒ*_S_ = 1 (Figure 2B). SNPs that were below this median frequency were interpreted as intraspecific variation within this overall homogenous isolate (Figure 2B). The homogeneity of CpGV-S was further confirmed by the absence of CpGV-M in CpGV-S, since all CpGV-M-specific SNPs had no frequencies (*ƒ*_M_ = 1) (Figure 2B).

In CpGV-0006, the frequencies of the CpGV-S-specific SNP positions were used in the same way to measure the presence of CpGV-S. Here, a ratio of about 67.8% CpGV-S was calculated based on the median frequencies of CpGV-S-specific SNPs (Figure 2C). The difference of the CpGV-S-specific portion of *ƒ*_S_ = 0.678 explained variability in CpGV-0006, which was considered as the hidden portion of CpGV-M, was calculated to be *ƒ*_M_ = 1 − *ƒ*_S_ = 0.322. Based on SNP frequency patterns and quantitative SNP analysis, CpGV-M and CpGV-S were considered highly homogenous isolates with minor possible internal genotypic variation represented by the scattering CpGV-M (Figure 2A) and CpGV-S (Figure 2B) specific SNP frequencies. In CpGV-0006, a ratio of about 67.8% CpGV-S was calculated based on the average frequencies of CpGV-S-specific SNPs (Figure 2C). The purity of isolates CpGV-M and CpGV-S was confirmed by the SNP analysis using CpGV-E2 as a alternative reference (Figure 2D–F). Here the 300 detected positions composed of 113 CpGV-S, 82 CpGV-M and 105 group-specific SNP positions (Figure 2D–F). For isolate CpGV-M, the relative frequencies of the isolate and group-specific SNPs were *ƒ*_M_ = 1, *ƒ*_S_ = 0 and *ƒ*_M+S_ = 1 (Figure 2D), reflecting the presence of only CpGV-M within the sequenced sample. Furthermore, the purity of isolate CpGV-S was confirmed by mapping CpGV-S reads against reference CpGV-E2, which resulted in relative frequencies of *ƒ*_M_ = 0, *ƒ*_S_ = 1 and *ƒ*_M+S_ = 1 (Figure 2E). In CpGV-0006, the median SNP frequencies were calculated with *ƒ*_M_ = 0.291, *ƒ*_S_ = 0.676 and *ƒ*_M+S_ = 1 (Figure 2F). Thus, the medians of *ƒ*_M_ and *ƒ*s explained 96.7% of the genetic composition and confirmed the presence of genotypes highly similar to CpGV-S (genome group E) and CpGV-M (genome group A) in CpGV-0006 in a ratio of about 2:1 (Figure 2F).

## 4. Discussion

In this study, we present a newly developed approach in combination with the bacsnp tool to determine the specificity of SNPs positions across sequenced baculovirus samples. It allows determining the intraspecific genetic variability of a single baculovirus isolate and follows the hypothesis that SNP positions with similar frequencies represent genotypes of equal frequency, and therefore, can be used for determination and characterization of isolates homo- and heterogeneity [1,6]. As for the ultra-deep sequenced isolate Autographa californica multiple nucleopolyhedrovirus WP10, where a single sample was investigated thoroughly, SNP positions were grouped by k-mean clustering into four clusters representing a significant variability within this isolate; a rather non-clonal replication of a baculovirus isolate was underlined [1]. Today, the mapping of reads against their own consensus sequence is a well-established method and allows the detection of intraspecific variation within sequenced baculovirus samples, such as Operophtera brumata nucleopolyhedrovirus [9], Erinnyis ello granulovirus [20] or Phthorimaea operculella granulovirus [10]; the latter two compare the genetic variation between sequenced isolates. Overall, genetic variation within mainly homogenous isolates was described in these sequenced samples and was confirmed for CpGV-M and CpGV-S in this study. Especially, this was visible for CpGV-M, which appeared homogenous but with minor variation that could be recognized by the pattern of CpGV-M-specific SNP positions with frequencies below 75%. This internal variation reoccurred partially in the mapping of CpGV-M reads against CpGV-E2 as reference. A similar assumption could be made for CpGV-S, which appeared homogenous with minor internal variation. Although the reference sequence of CpGV-M was assembled and determined from sequencing data that allowed only an average read depth of 17.23 [5], the sequence was considered as reliable and sufficiently reflected the majority of the viral population of CpGV-M. This was concluded since no SNP position was entirely different to the reference and therefore indicated no major sequencing or assembly errors at the date of consensus sequence creation.

The approach of self-mapping of reads against their own consensus sequence was impeded for isolate CpGV-0006 in downstream analyses when CpGV-0006 was initially found to be a distinct mixture of about two-thirds CpGV-S and one-third CpGV-M [6]. Here, the need for a method that omits the generation of a consensus sequence, which could render the genetic composition of CpGV-0006 only with ambiguous nucleotides but could not reflect any mixed ratios, became obvious. When applying the new bacsnp method, the assembly of reference sequences was not performed and instead reference sequences obtained from GenBank were used, irrespective of their correctness and completeness. Specificities of SNPs were called according to their frequencies only and allowed the calculation of the same ratio of CpGV-M and CpGV-S based on 277 or 300 SNP positions. From the set of 277 SNP positions found by the de novo approach, 233 positions were re-identified from those found by aligning consensuses of five different CpGV isolates, including CpGV-M and CpGV-S [5]. The remaining 42 positions that were not identified previously are part of the internal variations of these two isolates that were not reflected by their consensus sequences [5].

By analyzing SNP data with the bacsnp tool, it can be chosen if the analysis should be conducted on the frequencies of the first, second or third nucleotide alternative, which can be relevant in case more than two nucleotides were occurring at SNP positions. Based on the CpGV example of this study, only two nucleotides were identified at each SNP position after filtering the SNP frequency data. This finding is supported by the alignment of CpGV-M, CpGV-S and CpGV-E2 that did not exceed the presence of two different nucleotides at each found SNP position [5].

The deciphering and differentiation of two or multiple dominant genotypes within a sequenced baculovirus sample, as it was the case for CpGV-0006, is not always possible, because some isolates have been found to be highly variable in their genotype composition [12]. This was also the case for CpGV-E2, whose composition in genotypes was described to be highly heterogenous and complex and whose SNPs do not allow a clear differentiation [12]. However, the strength and the aim of bacsnp and its related workflow is the evaluation of a sequenced baculovirus population’s heterogeneity that genotype population studies or the analysis of biological functions of relevant individual SNPs can build on. For the correct detection of occurring SNPs, the choice of the reference sequence is a relevant and sometimes crucial factor that needs to be decided before the analysis is carried out. A closely related reference sequence at the species level is a prerequisite to prevent mismatching of reads during the assembly and detection of only true SNP positions. The forced mapping of reads to an unrelated reference sequence would not make any biological sense and would allocate numerous false variable nucleotide positions. A similar problem occurred in the repeat regions of this study when read mapping was ambiguous. In order to avoid any read mapping and assembly-related problems, the reference sequence for conducting the de novo detection of SNPs needs to be closely related (same baculovirus species) to the analyzed sample, as this was the case for CpGV-M and CpGV-E2, both being more than 98% identical in their nucleotide sequence and both belonging to the *Cydia pomonella granulovirus* species [5].

For CpGV-M and CpGV-E2 that belong to different genome groups (A and B, respectively) within their species, the independence of the reference sequence on the species level was underlined. Since the reference CpGV-M was part of the sequenced samples of CpGV-M and CpGV-0006, its presence cannot be interpreted intuitively from the analysis since its specific SNPs are missing in the plots. However, the proportion of CpGV-M in CpGV-0006 was determined by calculation as 32%, since it represented the missing part to the 68% that were explained by CpGV-S.

In case of using CpGV-E2 as a mapping reference, which is neither part of CpGV-M, CpGV-S nor CpGV-006, both the CpGV-M- and CpGV-S-specific SNP positions were clearly visible and interpretable. Most importantly indeed, both analyses resulted in very similar calculated ratios of CpGV-M and CpGV-S in the CpGV-M, CpGV-S and CpGV-006, independently from the used reference.

With the consensus sequence free method and newly developed bacsnp tool programmed in R, a rapid and easy-to-use downstream protocol is provided to evaluate the heterogeneity or homogeneity of sequenced baculovirus isolates. Furthermore, this method was developed to work for at least two to several isolates and allows cross-evaluating the presence of certain genotypes in each analyzed sample. The decision of whether a consensus sequence should be assembled from sequencing data of a given isolate is recommended to be done when the homogeneity and the absence of a mixture have been confirmed.

## Figures and Tables

**Table 1:** Result of the genome sequencing of isolates CpGV-M, CpGV-S and CpGV-0006 using short-read Illumina sequencing. Paired-end reads were 151 bp long.

Isolate	No. Reads		Paired/Unpaired (%)			Mapped to Reference ^b^ (%)	
	Total	Quality Filtered ^a^				CpGV-M	CpGV-E2
CpGV-M	3,886,630	3,644,161	95.7	/	4.3	99.6	99.8
CpGV-S	3,595,502	3,346,909	95.2	/	4.8	89.1	89.1
CpGV-0006	1,508,218	1,424,605	96.2	/	3.8	99.2	99.3

^a^ Adapter trimming and quality filtering with Phred quality score ≥30 (base-call accuracy 99.9%). ^b^ Percentage refers to number of quality filtered reads.

**Table 2:** Mean frequencies of the reference (*ƒ*_ref_) and three alternative nucleotides (*ƒ*_rel1_, *ƒ*_rel2_ and *ƒ*_rel3_). Frequencies were calculated from the 284 SNP positions referring to the mappings of short-read sequencing data of CpGV-M, CpGV-S and CpGV-0006 against CpGV-M reference sequence. Given are the arithmetic means and standard deviation.

Isolate	Mean Frequency and Standard Deviation				*ƒ*_ref_ + *ƒ*_alt1_	*ƒ*_alt2_ + *ƒ*_alt3_
	*ƒ*_ref_	*ƒ*_alt1_	*ƒ*_alt2_	*ƒ*_alt3_		
CpGV-M	0.970 ± 0.092	0.028 ± 0.092	0.001 ± 0.002	0.001 ± 0.001	0.998	0.002
CpGV-S	0.087 ± 0.252	0.910 ± 0.254	0.002 ± 0.015	0.001 ± 0.001	0.997	0.003
CpGV-0006	0.394 ± 0.199	0.605 ± 0.199	0.001 ± 0.002	0.000 ± 0.001	0.998	0.002
